# Alcohol-related problems and primary care in Portugal: the state of the art

**DOI:** 10.1186/1940-0640-7-S1-A31

**Published:** 2012-10-09

**Authors:** Cristina Ribeiro

**Affiliations:** 1 Department of Treatment and Rehabilitation, Institute on Drugs and Drug Addiction, Lisbon, Portugal

## 

Portugal has the highest levels of alcohol consumption in the world and the highest rates of alcohol-related problems. The Portuguese government’s National Plan to Reduce Alcohol Related Problems includes the development of projects that provide training to health professionals in screening and intervention for alcohol use disorders, particularly for professionals who work in primary care. Primary care professionals are the first level of assistance in Portugal’s national health care system and have ongoing contact with people who use primary health centers. High consumption of alcohol is responsible for a number off physical, psychological, and social problems. The high prevalence of excessive alcohol consumption and its high economic, social, and health costs justify the implementation of such projects to address alcohol-related problems. Objectives of the national plan include identifying needs specific to primary care settings; designing a training program tailored to primary care center needs; defining priorities (e.g., providing services and building infrastructure to optimize hazardous and harmful alcohol use management and providing monitoring to ensure adherence to protocols that consider clinical referral procedures and specific indicators); ensuring both undergraduate and postgraduate training; and identifying organizational and political components critical to the successful implementation and dissemination of alcohol screening and brief intervention in primary care. We show preliminary results of the plan and the importance of addressing alcohol-relate problems at the national and international level to facilitate sharing of knowledge and standardizing of evidence-based practice between countries.

